# Immune persistence 17 to 20 years after primary vaccination with recombination hepatitis B vaccine (CHO) and the effect of booster dose vaccination

**DOI:** 10.1186/s12879-019-4134-9

**Published:** 2019-05-30

**Authors:** Yu-Liang Zhao, Bi-Hua Han, Xin-Jiang Zhang, Lu-Lu Pan, Hai-Song Zhou, Zhao Gao, Zhi-Yong Hao, Zhi-Wei Wu, Tian-Li Ma, Feng Wang, Qi Li, Sheng-Li Bi, Jing-Chen Ma

**Affiliations:** 1 Institute for Vaccine Clinical Research, Hebei Province Center for Disease Control and Prevention, 97 Huai’an East Road, Yuhua District, Shijiazhuang, 050021 Hebei Province People’s Republic of China; 2 Zhengding County Center for Disease Control and Prevention, Zhengding, 050800 People’s Republic of China; 3 Institute for Viral Disease Control and Prevention, China Center for Disease Control and Prevention, Changing District, Beijing, 100052 People’s Republic of China

**Keywords:** Immune persistence, Hepatitis B virus, Vaccine, Booster

## Abstract

**Background:**

To assess the immune persistence conferred by a Chinese hamster ovary (CHO)-derived hepatitis B vaccine (HepB) 17 to 20 years after primary immunization during early life.

**Methods:**

Participants born between 1997 and 1999 who received a full course of primary vaccination with HepB (CHO) and who had no experience with booster vaccination were enrolled. Blood samples were required from each participant for measurement of hepatitis B surface antibody (anti-HBs), surface antigen and core antibody levels. For those who possessed an anti-HBs antibody < 10 mIU/mL, a single dose of HepB was administered, and 30 days later, serum specimens were collected to assess the booster effects.

**Results:**

A total of 1352 participants were included in this study. Of these, 1007 (74.5%) participants could retain an anti-HBs antibody ≥10 mIU/mL, with a geometric mean concentration (GMC) of 57.4 mIU/mL. HBsAg was detected in six participants, resulting in a HBsAg carrier rate of 0.4% (6/1352). Of those participants with anti-HBs antibodies < 10 mIU/mL, after a challenge dose, 231 (93.1%) presented an anti-HBs antibody ≥10 mIU/mL, with a GMC of 368.7 mIU/mL. A significant increase in the anti-HBs positive rate (≥ 10 mIU/mL) after challenge was observed in participants with anti-HBs antibodies between 2.5 and 10 mIU/mL and participants boosted with HepB (CHO), rather than those with anti-HBs antibodies < 2.5 mIU/mL and those boosted with HepB (SC).

**Conclusion:**

Since satisfactory immune protection against HBV infection conferred by primary vaccination administered 17–20 years ago was demonstrated, there is currently no urgent need for booster immunization.

## Background

Hepatitis B virus (HBV) is one of the leading causes of infectious diseases and a major public health problem in China. The hepatitis B vaccine (HepB) is the most effective means to control the spread of HBV. In 2002, HepB in China was officially integrated into the China National Immunization Plan, and newborns underwent obligatory vaccination at 0, 1 and 6 months of age. A satisfactory immune response was observed among 90 to 95% of healthy infants, whereas 5 to 10% of the population showed no response or a weak response [1–3]. Moreover, for the time being, a decline in hepatitis B surface antibody (anti-HBs) level was observed among those infants with a satisfactory immune response after initial injection. Theoretically, anti-HBs < 10 mIU/mL is considered the threshold for effective protection against HBV infection. To control HBV infection, monitoring the long-term persistence after initial injection in real time to determine the timing for a booster dose is important.

In the 1980s, Zhengding County, Hebei Province, China, was selected as the study site for the clinical evaluation of plasma-derived HepB [4]. Since January 1, 1997, newborns were vaccinated with Chinese hamster ovary (CHO)-derived HepB. To evaluate the immune persistence 17 to 20 years after primary vaccination with CHO-derived HepB and the effects of a booster dose vaccination, we conducted this study between August 2017 and September 2018.

## Methods

### Study cohort and study design

Universal HepB immunization in newborns began in 1986 in the community. From then on, a database about the assessment of the immunity efficacy of HepB was established in which data about primary immunization were recorded. Between 1997 and 2001, a CHO-derived HepB with a dosage of 10 μg/mL was used for the newborns according to the 0, 1, and 6 month schedule. In total, 92.9% of individuals maintained positive for anti-HBs in the cross-sectional survey in May 1999. By screening the historical database, participants born between 1997 and 1999 living in seven townships in Zhengding County who completed the full course primary vaccination were enrolled in this study. In the present study, a questionnaire, including name, sex, date of birth, and history of HepB booster vaccination, was completed, and those who underwent a booster dose before this study were excluded from the final analysis. A blood sample was collected from each participant who provided written informed consent, and the serum was isolated aseptically and stored at − 20 °C until testing.

Participants who were hepatitis B surface antigen (HBsAg)- and hepatitis B core antibody (anti-HBc)-negative and anti-HBs < 10 mIU/mL were randomly assigned to two groups using the random numbers 1 and 2, receiving a booster dose of HepB (20 μg/ml) derived from either *Saccharomyces cerevisiae* (SC) (Shenzhen Kangtai Biological Products Co., Ltd., Shenzhen, China) or CHO (Genetech Biotechnology, Huabei Pharmaceutical Co., Ltd., Shijiazhuang, China). Blood samples were collected 30 days after boosting to determine the potential immunological response.

This study was reviewed and approved by the Institutional Review Board (IRB) of the Hebei Center for Disease Control and Prevention (CDC). Written informed consent was obtained from each participant for personal information and blood sample collection. The study was performed in accordance with the ethical standards established in the 1964 Declaration of Helsinki and its later amendments.

### Laboratory assay

A batch test for virological and immunological biomarkers of HBV was performed in the reference laboratory at the Institute for Viral Disease, China Center for Disease Control and Prevention (Beijing) upon the completion of follow-up. Abbott EIA AxSYM (Abbott, Abbott Park, IL, USA) was used for the detection of HBsAg, anti-HBs, and anti-HBc. According to protocols provided by the manufacturer, positive and negative cutoffs were calculated with the positive and negative controls as required by the diagnostic kits. HBsAg < 0.05 IU/mL was considered reactive. The minimum detection limit of anti-HBs was 2.5 mIU/mL.

### Statistical analysis

All data were double entered into custom-made data entry programs based on Epidata 3.1. The data management programs included range and consistency checks. An SPSS program (IBM Corporation, Armonk, NY, USA) was used for statistical analysis. Antibody seroprotection rate (SPR), geometric mean concentrations (GMCs) and their 95% confidence intervals (CIs) were calculated. Antibody concentrations were logarithmically converted to allow for assessment of GMCs. For continuous outcome comparisons, Student’s t test or the Mann–Whitney U test was performed, and for dichotomous outcomes, the chi square or Fisher exact test was implemented. The Mantel-Haenszel test was conducted for stratification analysis. Seroprotection was defined as an anti-HBs ≥ 10 mIU/mL. Time after vaccination was defined as the interval between the last visit (time for blood collection during the cross-sectional survey in the present study) and completion of the full primary course. HBV infection was defined as positive for HBsAg or/and anti-HBc. A *p*-value < 0.05 was considered statistically significant.

## Result*s*

### Baseline characteristics of enrolled participants

Between 1997 and 1999, 2436 newborns who received a full course of primary vaccination were initially recruited in the study cohort, and 1551 (63.7%) participants were followed in 2017. Of the 1551 individuals, 199 individuals with a history of booster vaccination were excluded, and 1352 (55.5%) participants were included in the final analysis. Of the 1352 participants, 616 (45.6%) were male, and the average age of the enrolled participants was 19.3 years (95% CI: 19.2–19.3). All participants completed primary vaccination at day 187.2 (95% CI: 185.4–189.0) after birth, resulting in an average of 18.8 (95% CI: 18.7–18.8) follow-up years.

### Long-term protection against HBV infection

After measurement of the anti-HBs antibody, 1007 (74.5%) participants had an anti-HBs antibody level ≥ 10 mIU/mL, with a GMC of 57.4 mIU/mL (95% CI: 50.4–66.0 mIU/mL) at 17 to 20 years after the initial dose of HBV vaccine. The seroprotection rates were 74.0 and 75.0% in the female and male subgroups, respectively (*p >* 0.05); moreover, a similar GMC was observed in females (55.1 mIU/mL, 95% CI: 45.6–66.7 mIU/mL) and males (60.9 mIU/mL, 95% CI: 49.9–74.4 mIU/mL) (p > 0.05). A considerable percentage (46.6%; 630/1352) of participants possessed an anti-HBs antibody ≥100 mIU/mL. Of these, 167 participants had an anti-HBs antibody ≥1000 mIU/mL.

During the long-term follow-up, of 1352 participants, 18 (1.3%) experienced HBV infection. HBsAg was detected in 6 participants, all of whom were positive for anti-HBc, and the HBsAg positive rate was 0.4% (6/1352). The other 12 participants did not carry HBV, which led to an anti-HBc positive rate of 0.9% (12/1352).

### Recall responses after the booster

**Fig. 1:**
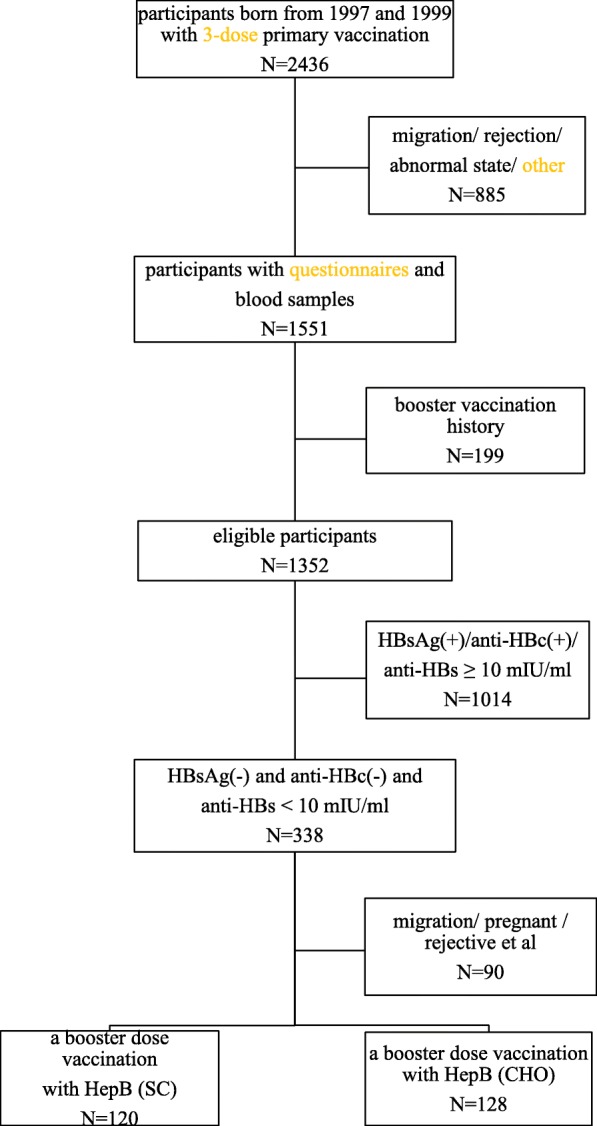
Assembly chart of participants through the study

Among the 338 participants who were HBsAg- and anti-HBc-negative, with anti-HBs antibody < 10 mIU/mL at 17 to 20 years, 248 (73.4%) participants provided informed consent and received a single booster dose of HBV vaccine. Of these, 128 participants received a single dose of HepB (CHO), while another 120 participants were injected with a single dose of HepB (SC) (Fig. 1).

One month after administration of a single dose of HBV vaccine, 93.1% (231/248) of vaccinees developed detectable anti-HBs antibody with a GMC of 368.7 mIU/mL (95% CI: 278.7–487.8 mIU/mL). The majority (78.2%) of vaccinees displayed a dramatic increase in anti-HBs titers that was ≥10-fold (Table 1). Similar to the abovementioned results, a significant difference was not detected in the anti-HBs antibody level between females and males, neither seroconversion rate (95.0% vs 90.8%), nor antibody concentrations (450.3mIU/mL vs 284.3 mIU/mL) (*p* > 0.05).

**Table 1:** Distribution of anti-HBs in individuals 30 days after booster vaccination

Anti-HBs(mIU/mL)	No.	Constituent ratio (%)	GMC (mIU/mL)	95% CI
< 10	17	6.9	1.5	0.9–2.7
10~100	37	14.9	39.3	31.2–48.9
100~1000	115	46.4	379.9	333.6–432.7
≥1000	79	31.8	3261.7	2643.9–3983.8

**Fig. 2:**
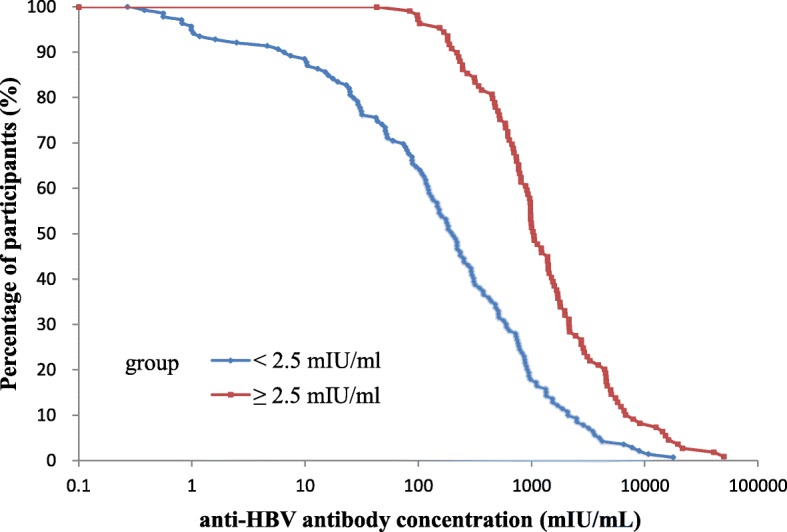
Reverse cumulative distribution curves for individual post-booster anti-HBV concentrations in pre-booster anti-HBs concentrations < 2.5 mIU/ml and ≥ 2.5 mIU/ml

When those participants who received the booster dose of HBV vaccine were further stratified into two subgroups by the minimum detection limit of Abbott reagent of 2.5 mIU/mL, a statistically significant difference was found between participants with pre-booster anti-HBs antibody concentration < 2.5 mIU/mL and participants with pre-booster anti-HBs antibody concentration ≥ 2.5 mIU/mL, either for the seroconversion rate [87.8% (122/139) vs 100% (109/109)] (*p* < 0.001) or for the GMC [144.0 mIU/mL (95% CI: 97.5–214.9 mIU/mL) vs 1212.0 mIU/mL (95% CI: 934.5–1571.8 mIU/mL)] (p < 0.001) (Fig. 2).

**Fig. 3:**
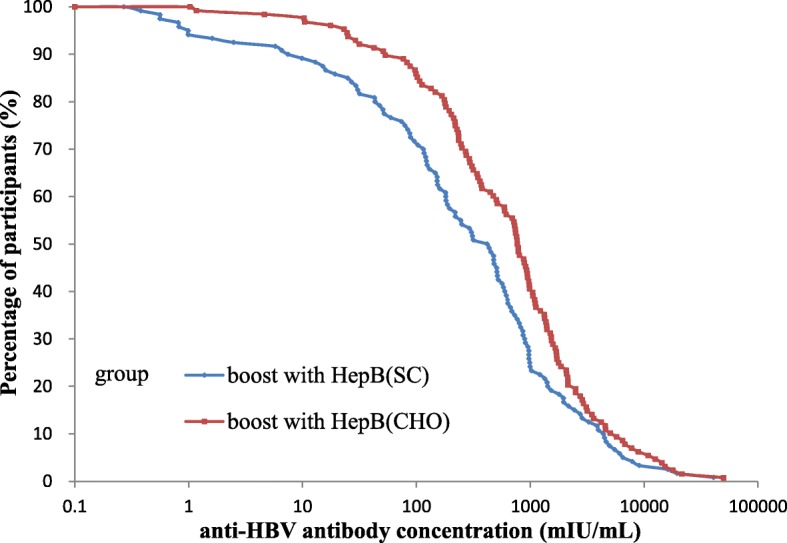
Reverse cumulative distribution curves for individual post-booster anti-HBs concentrations in the HepB (CHO) and HepB (SC) groups

When boostered participants were aggregated into the HepB (CHO) challenge group and the HepB (SC) challenge group, the average age and gender composition were comparable in the two groups (19.2-years vs. 19.3-years, *p* > 0.05; gender composition ratio: 1.0:1.5 vs. 1.0:1.1; p > 0.05). The difference in the pre-booster anti-HBs titers was not statistically significant in the two groups [HepB (CHO): 3.1 mIU/mL (95% CI: 2.6–3.6 mIU/mL); HepB (SC): 3.2 (95% CI: 2.6–3.7 mIU/mL); p > 0.05]. The seroconversion rates were 97.7% (125/128) and 88.3% (106/120) in the two groups (*p* < 0.05). A higher anti-HBs concentration was observed in the HepB (CHO) challenge group (578.2 mIU/mL; 95% CI: 419.9–804.3 mIU/mL) rather than in the HepB (SC) challenge group (225.9 mIU/mL; 95% CI: 145.5–354.2 mIU/mL), and a significant difference was reached (p < 0.05) (Fig. 3). After adjusting for the risk factor for pre-booster anti-HBs titers, the difference in the seroconversion rates between the two groups was statistically significant (*p* = 0.006).

## Discussion

In this study, a seroprotection rate of 74.5%, with a GMC of 57.4 mIU/mL, was observed in a longitudinal birth cohort who completed the full primary course of HBV immunization 17 to 20 years ago. Our previous cross-sectional surveys based on the same cohort reported a seroprotection rate of 76.8% (GMC: 276 mIU/mL) and 74.1% (GMC: 209 mIU/mL) at 6 to 8 years and 10 to 12 years after completion of primary vaccination, respectively [5, 6]. Notably, for the time being, the anti-HBs seroprotection rates were quite stable compared to the decline in anti-HBs antibody concentrations. Several studies have reported the persistence of anti-HBs levels after primary vaccination and found that protective anti-HBs antibodies were maintained up to 20 years in approximately 37–77% of vaccinees [7–10]. The persistence of anti-HBs is related to a variety of factors, including the mother’s carrying status, regimens and type of vaccine used, time interval between the first and last injection of primary vaccination, and anti-HBs antibody peak level after full course of primary immunization [10–12].

A series of studies reported a satisfactory anti-HBs seroconversion, which was approximately 84.0 and 98.0%, after a booster dose was achieved [7, 12–16]. In our study, a dramatic increase in the anti-HBs antibody positive rate was measured. More than 90% of participants presented an anti-HBs antibody level ≥ 10 mIU/mL. A robust anamnestic response even in those with pre-challenge anti-HBs antibody < 10 mIU/mL was thus demonstrated. However, an anti-HBs antibody level in 6.9% participants was still less than 10 mIU/mL although it had increased multifold. The likely explanation is the genetic non-response that also occurs during the primary immunization or the decay with time of immunization memory, considering that the anti-HBs levels of the majority of these participants were less than 10 mIU/mL in previous cross-sectional surveys based on the same cohort. It would be interesting to perform genetic analysis on these participants to understand the potential association between gene mutation and response to HBV vaccination.

Theoretically, primary immunization is the basis of immunity established in human bodies, and a booster effect depends on the pre-challenge antibody level. For people with pre-challenge anti-HBs levels < 10 mIU/mL, after boosting, a higher antibody level could be expected in vaccinees with low anti-HBs levels at pre-challenge compared to that in those vaccinees with undetectable anti-HBs (anti-HBs antibody level < the lower limit of detection) [14, 17]. Our study demonstrated again that a significant increase in seroprotection was found in participants with a pre-challenge anti-HBs antibody between 2.5–10 mIU/mL in comparison with those participants with an anti-HBs antibody < 2.5 mIU/mL. In addition, different booster effects were measured between participants boostered with HepB (CHO) and participants boostered with HepB (SC). The former displayed a better response to boosting. Though controversial conclusions on immune response to a challenge dose were generated from different studies [18–20], a higher immune response to primary immunization was always conferred by HepB (CHO) rather than HepB (SC). The possible explanation is the different varieties of vaccines between primary immunization and booster dose. Another study showed that the possibility of the potential mismatch between the antibody elicited by HepB (SC) and the antigen derived from blood donors, which is coated on the reagent plate, cannot be excluded and may affect the sensitivity of the detection [21].

There are several limitations in our study. First, serological surveys were not conducted every year, and the occurrence of a booster due to natural HBV infection could not be detected and eventually could not be excluded from the current analysis. From this point, the persistence of immunity demonstrated in this study was conferred by both vaccine immunization and natural infection. However, because HBV is transmitted through fluids, mainly through sexual transmission, teenagers living in rural areas usually have lower chance of exposure to HBV. Thus, natural infection did not contribute too much to the maintenance of immunity. Second, applying different immunoassay systems with incomparable sensitivity over a long time period is often inevitable and may damage the consistency of serological data. However, in comparison with the seroprotection rate and anti-HBs antibody concentration tested at 6 to 8 years and 10 to 12 years after completion of primary vaccination, a trend of continuous and stable decline, in stead of fluctuation of immunity, was observed. To a certain extent, there was comparability between different generations of Abbott assay systems. Finally, the history of booster immunization was obtained from the questionnaire, in which recall bias might exist.

The demand for booster and the timing of boosters after the completion of primary immunization are always controversial. Some studies concluded that booster vaccination was necessary when antibodies fell below the protective level threshold to reach an effective antibody level against HBV infections [15, 16, 22, 23]. However, other studies indicated that there was no evidence to support booster vaccination thus far [12, 24–26]. In particular, the cellular immune memory could be determined from majority of vaccinees whose anti-HBs antibody level was lower than 10 mIU/mL, or even absent [27]. In 2000, the European Consensus Group on Hepatitis B Immunity issued a statement that there were no data supporting a need for booster doses of HepB in immunocompetent individuals who had responded to a primary course [28]. More recently, a WHO position paper also noted that there was no evidence supporting the need for a booster dose of HepB in routine immunization programs [29]. Considering a dramatic and continued reduction in the HBsAg carrier rate in this age group of the entire population in China [30, 31], together with findings from our study, we conclude that there is currently no urgent need for booster immunization.

## Conclusion

A protective anti-HBs antibody level conferred by primary vaccination administered 17–20 years ago was maintained in approximately three-quarters of the vaccinees, and a satisfactory anamnestic response was observed in the majority of individuals with a pre-booster anti-HBs antibody level < 10 mIU/mL. Pre-booster anti-HBs antibody concentrations and type of vaccines were factors associated with post-booster anti-HBs antibody levels.

## Data Availability

The datasets generated and analyzed during the current study are not publicly available because the personal information of the participants is confidential but are available from the corresponding author on reasonable request.

## Abbreviations

anti-HBc: Hepatitis B core antibody
Anti-HBs: Hepatitis B surface antibody
CHO: Chinese hamster ovary
GMC: Geometric mean concentration
HBsAg: Hepatitis B surface antigen
HBV: Hepatitis B virus
HepB: Hepatitis B vaccine
SC: *Saccharomyces cerevisiae*
